# Hydrogen Peroxide-Induced Root Ca^2+^ and K^+^ Fluxes Correlate with Salt Tolerance in Cereals: Towards the Cell-Based Phenotyping

**DOI:** 10.3390/ijms19030702

**Published:** 2018-03-01

**Authors:** Haiyang Wang, Lana Shabala, Meixue Zhou, Sergey Shabala

**Affiliations:** School of Land and Food, University of Tasmania, Hobart, Tasmania 7001, Australia; Haiyang.Wang@utas.edu.au (H.W.); L.Shabala@utas.edu.au (L.S.); Meixue.Zhou@utas.edu.au (M.Z.)

**Keywords:** ion flux, reactive oxygen species, barley, wheat, oxidative stress, salinity stress, microelectrode ion flux estimation

## Abstract

Salinity stress-induced production of reactive oxygen species (ROS) and associated oxidative damage is one of the major factors limiting crop production in saline soils. However, the causal link between ROS production and stress tolerance is not as straightforward as one may expect, as ROS may also play an important signaling role in plant adaptive responses. In this study, the causal relationship between salinity and oxidative stress tolerance in two cereal crops—barley (*Hordeum vulgare*) and wheat (*Triticum aestivum*)—was investigated by measuring the magnitude of ROS-induced net K^+^ and Ca^2+^ fluxes from various root tissues and correlating them with overall whole-plant responses to salinity. We have found that the association between flux responses to oxidative stress and salinity stress tolerance was highly tissue specific, and was also dependent on the type of ROS applied. No correlation was found between root responses to hydroxyl radicals and the salinity tolerance. However, when oxidative stress was administered via H_2_O_2_ treatment, a significant positive correlation was found for the magnitude of ROS-induced K^+^ efflux and Ca^2+^ uptake in barley and the overall salinity stress tolerance, but only for mature zone and not the root apex. The same trends were found for wheat. These results indicate high tissue specificity of root ion fluxes response to ROS and suggest that measuring the magnitude of H_2_O_2_-induced net K^+^ and Ca^2+^ fluxes from mature root zone may be used as a tool for cell-based phenotyping in breeding programs aimed to improve salinity stress tolerance in cereals.

## 1. Introduction

Salinity stress is one of the major environmental constraints limiting crop production worldwide that results in massive economic penalties, especially in arid and semi-arid regions [1,2,3]. Because of this, plant breeding for salt tolerance is considered to be a major avenue to improve crop production in salt affected regions [4]. According to the classical view, two major components—osmotic stress and specific ion toxicity—limit plant growth in saline soils [5]. Unsurprisingly, in the past decades, many attempts have been made to target these two components in plant breeding programs. The major efforts were focused on either improving plant capacity to exclude Na^+^ from uptake by targeting *SOS1* [6,7,8] and *HKT1* [9,10,11] genes, or increasing de novo synthesis of organic osmolytes for osmotic adjustment [12,13,14]. However, none of these approaches has resulted in truly tolerant crops in the farmers’ fields, and even the best performing genotypes created showed a 50% of yield loss when grown under saline conditions [9].

In addition to osmotic and ionic component of the salt stress, one of the reasons for the above detrimental effects of salinity on plant growth is the overproduction and accumulation of reactive oxygen species (ROS) under saline condition [15,16]. The increasing level of ROS in a green tissue under saline condition results from the impairment of the photosynthetic apparatus and a limited capability for CO_2_ assimilation in a conjunction with plant’s inability to fully utilize light captured by photosynthetic pigments [17,18]. However, leaf is not the only site of ROS generation, as the latter species can also be produced in root tissues under saline condition [15,19,20,21,22]. In Arabidopsis roots, increasing hydroxyl radicals (^●^OH) [23] and H_2_O_2_ [24] levels were observed under salt stress. Accumulation of NaCl-induced H_2_O_2_ was also observed in rice [25] and pea roots [26].

When ROS are accumulated in excessive quantities in plant tissues, significant damage to key macromolecules and cellular structures occurs [27,28]. However, the disturbance to cell metabolism (and associated growth penalties) may occur well before this damage is observed. ROS generation in root tissues occurs rapidly in response to salt stimuli and leads to the activation of a broad range of ion channels including Na^+^-permeable non-selective cation channels (NSCCs) and outward rectifying efflux K^+^ channels (GORK). This results in a disequilibrium of the cytosolic ions pools and a perturbation of cell metabolic processes. When the cytosolic K^+^/Na^+^ ratio is shifted beyond some critical threshold, the cell can undergo a programmed cell death (PCD) [29,30]. Taken together, these findings have prompted an idea of improving salinity stress tolerance via enhancing plant antioxidant activity [31,32]. However, despite numerous attempts [33,34,35], the practical outcomes of this approach are rather modest [36,37].

One of the reasons for the above failure to improve plant stress tolerance via constitutive expression of enzymatic antioxidants is the fact that ROS also play an important signaling role in plant adaptive and developmental responses [38]. Moderate level of ROS is essential for signaling pathways mediating a diverse range of physiological and developmental processes [15,38,39,40,41,42,43]. Therefore, scavenging ROS by constitutive expression of enzymatic antioxidants (AOs) may interfere with these processes and cause pleiotropic effects. As a result, the reported association between activity of AO enzymes and salinity stress tolerance is often controversial [44], and the entire concept “the higher the AO activity the better” does not hold in many cases [45,46,47].

ROS are known to activate Ca^2+^ and K^+^-permeable plasma membrane channels in root epidermis [48], resulting in elevated Ca^2+^ and depleted K^+^ pool in the cytosol, with a consequent disturbance to intracellular ion homeostasis. A pivotal importance of K^+^ retention under salinity stress is well known and has been widely reported to correlate positively with the overall salinity tolerance in roots of both barley and wheat, as well as many other species (reviewed by Shabala [49]). Elevation in the cytosolic free Ca^2+^ is also observed in response to a broad range of abiotic and biotic stimuli, and has long been considered an essential component of cell stress signaling mechanism [50,51,52]. In light of the above, and given the dual role of ROS and their involvement in multiple signaling transduction pathways [38], should salt tolerant species and genotypes be more or less sensitive to ROS? Is this sensitivity the same for all tissues, or does it show some specificity? Can the magnitude of the ROS-induced ion fluxes across the plasma membrane be used as a physiological marker in breeding programs to improve plant salinity stress tolerance? To the best of our knowledge, none of the previous studies has examined ROS-sensitivity of ion transporters in the context of tissue-specificity, or explored a causal link between two type of ROS applied and stress-induced changes in plant ionic homeostasis, in the context of salinity stress tolerance. This gap in our knowledge was addressed in this work.

In this study, we employed the non-invasive microelectrode ion flux estimation (MIFE) technique to address the above questions and investigate the correlation between oxidative stress-induced ion responses and plant’s overall salinity stress tolerance. The ultimate aim of this work is to develop the cell-based phenotyping approach that can then be employed by breeders for QTL mapping of these traits, in order to improve salinity stress tolerance in plant species.

## 2. Results

### 2.1. H_2_O_2_-Induced Ion Fluxes Are Dose-Dependent

**Figure 1:**
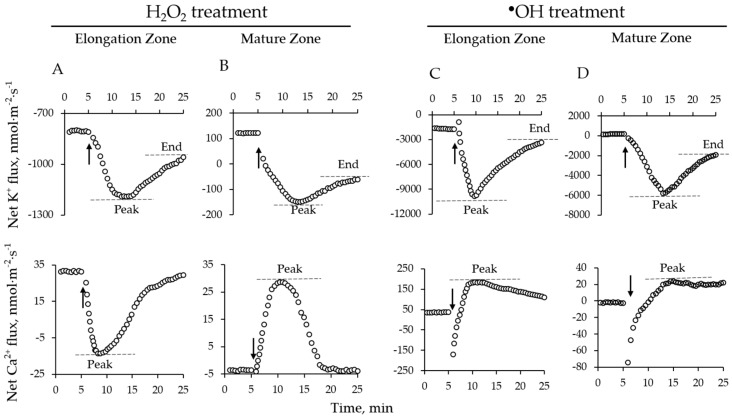
Descriptions (see inserts in each panel) of cereal root ion fluxes in response to H_2_O_2_ and hydroxyl radicals (^●^OH) in a single experiment. (**A**,**B**) Ion flux kinetics in root elongation zone (**A**) and mature zone (**B**) in response to H_2_O_2_; (**C**,**D**) Ion flux kinetics in root elongation zone (**C**) and mature zone (**D**) in response to ^●^OH. Two distinctive flux points were identified in kinetics of responses: peak value-identified as a maximum flux value measured after a treatment; end value-identified 20 min after the treatment application. An arrow in each panel represents when oxidative stress was imposed.

Two parameters were identified and analyzed from transient response curves (Figure 1). The first one was peak value, defined as the maximum flux value measured after the treatment; and the second was the end value, defined as a baseline flux 20 min after the treatment application.

**Figure 2:**
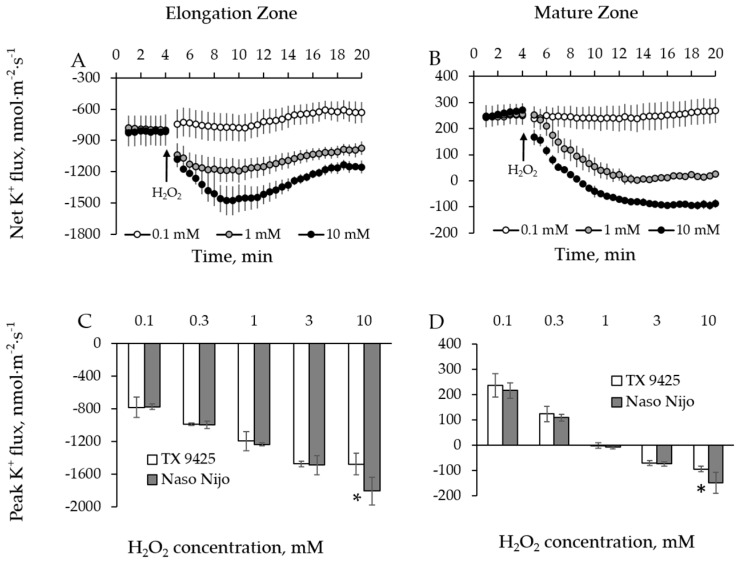
(**A**,**B**) Net K^+^ fluxes measured from barley variety TX 9425 root elongation zone (**A**)—about 1 mm from the root tip and mature zone (**B**)—about 30mm from the root tip with respective H_2_O_2_ concentrations. (**C**,**D**) Dose-dependency of H_2_O_2_-induced K^+^ fluxes from root elongation zone (**C**) and mature zone (**D**). Error bars are means ± SE (*n* = 6–8). Asterisks indicate statistically significant differences between two varieties (* *p* < 0.05, Student’s *t*-test). Responses from Naso Nijo were qualitatively similar to those shown for TX 9425.

**Figure 3:**
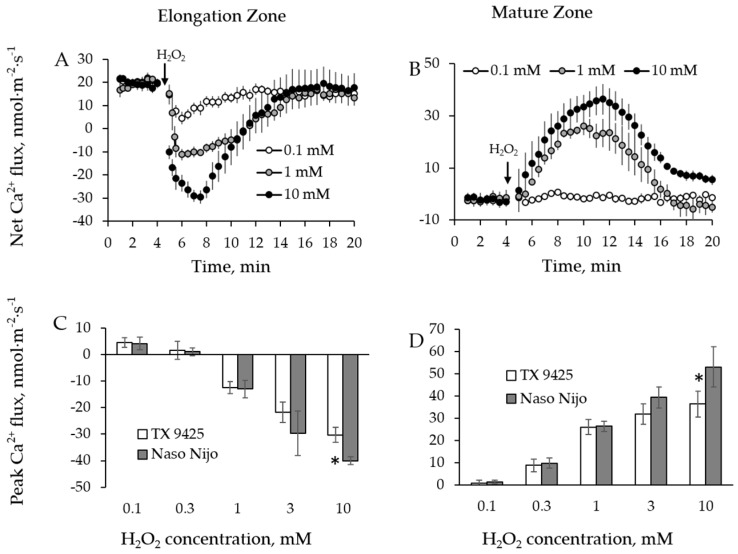
(**A**,**B**) Net Ca^2+^ fluxes measured from barley variety TX 9425 root elongation zone (**A**) and mature zone (**B**) with respective H_2_O_2_ concentrations. (**C**,**D**) Dose-dependency of H_2_O_2_-induced Ca^2+^ fluxes from root elongation zone (**C**) and mature zone (**D**). Error bars are means ± SE (*n* = 6–8). Asterisks indicate statistically significant differences between two varieties (* *p* < 0.05, Student’s *t*-test). Responses from Naso Nijo were qualitatively similar to those shown for TX 9425.

Two barley varieties (TX 9425, salinity tolerant; Naso Nijo, salinity sensitive) were used for optimizing the dosage of H_2_O_2_ treatment. Accordingly, TX 9425 and Naso Nijo roots were treated with 0.1, 0.3, 1, 3, and 10 mM H_2_O_2_ and ion fluxes data were acquired from both root mature and elongation zones for 15 min after application of H_2_O_2_. We found that, except for 0.1 mM, all the H_2_O_2_ concentrations triggered significant ion flux responses in both root zones (Figure 2A,B and Figure 3A,B). In the elongation root zone, an initial K^+^ efflux (negative flux values, Figure 2A) and Ca^2+^ uptake (positive flux values, Figure 3A) were observed. Application of H_2_O_2_ to the root led to a more intensive K^+^ efflux and a reduced Ca^2+^ influx (the latter turned to efflux when concentration of H_2_O_2_ was ≥1 mM) (Figure 2A and Figure 3A). In the mature root zone, the initial K^+^ uptake (Figure 2B) and Ca^2+^ efflux (Figure 3B) were observed. Application of H_2_O_2_ to the bath led to a dramatic K^+^ efflux and Ca^2+^ uptake (Figure 2B and Figure 3B). Ca^2+^ flux has returned to pre-stress level after reaching a peak (Figure 3A,B). Fluxes of K^+^, however, remained negative after reaching the respective peak (Figure 2A,B). The time required to reach a peak increased with an increase in H_2_O_2_ concentration (Figure 2A,B and Figure 3A,B).

The peak values for both Ca^2+^ and K^+^ fluxes showed a clear dose-dependency for H_2_O_2_ concentrations used (Figure 2C,D and Figure 3C,D). The biggest significant difference (*p* ˂ 0.05) in ion flux responses of contrasting varieties was observed at 10 mM H_2_O_2_ for both K^+^ (Figure 2C,D) and Ca^2+^ fluxes (Figure 3C,D). Accordingly, 10 mM H_2_O_2_ was chosen as the most suitable concentration for further experiments.

### 2.2. Genotypic Variation in H_2_O_2_-Induced Ca^2+^ and K^+^ Fluxes in Barley

**Figure 4:**
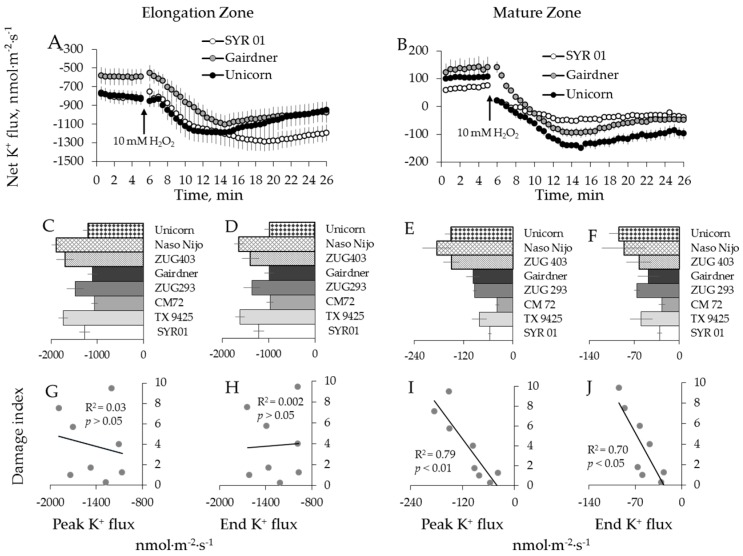
Kinetics of K^+^ fluxes from three representative barley varieties in response to 10 mM H_2_O_2_ treatment from both root elongation zone (**A**) and mature zone (**B**). Error bars are means ± SE (*n* = 6–8). (**C**,**D**,**G**,**H**) Peak (**C**) and end (**D**) K^+^ fluxes of eight barley varieties in response to 10 mM H_2_O_2_ and their correlation with damage index (**G**,**H**, respectively) in root elongation zone. (**E**,**F**,**I**,**J**) Peak (**E**) and end (**F**) K^+^ fluxes of eight barley varieties in response to 10 mM H_2_O_2_ and their correlation with damage index (**I**,**J**, respectively) in root mature zone.

**Figure 5:**
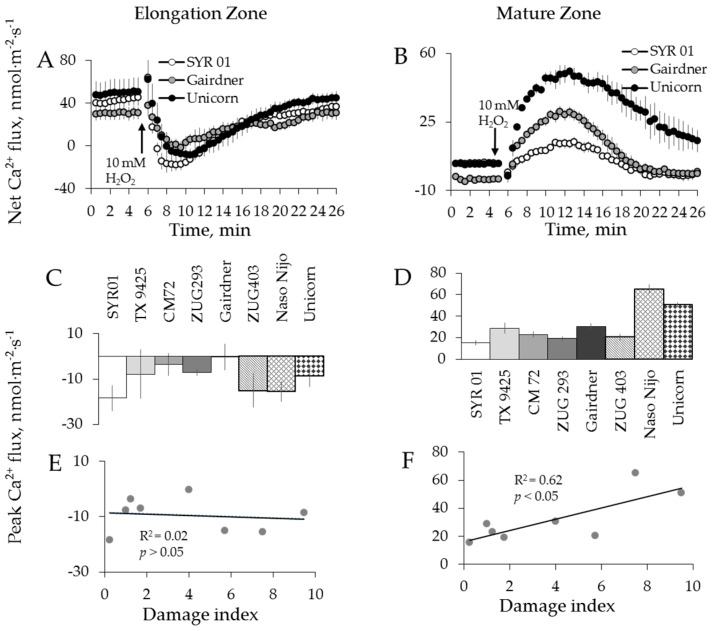
Kinetics of Ca^2+^ fluxes from three representative barley varieties in response to 10 mM H_2_O_2_ treatment from both root elongation zone (**A**) and mature zone (**B**). Error bars are means ± SE (*n* = 6–8). (**C**,**E**) Peak Ca^2+^ fluxes (**C**) of eight barley varieties in response to 10 mM H_2_O_2_ and their correlation with damage index (**E**) in root elongation zone. (**D**,**F**) Peak Ca^2+^ fluxes (**D**) of eight barley varieties in response to 10 mM H_2_O_2_ and their correlation with damage index (**F**) in root mature zone.

Once the optimal H_2_O_2_ concentration was chosen, eight barley varieties contrasting in their salt tolerance (see Table 1) were tested for their ability to maintain K^+^ and Ca^2+^ homeostasis under 10 mM H_2_O_2_ treatment (Figure 4 and Figure 5). The kinetics of K^+^ flux responses were qualitatively similar and the magnitudes were dramatically different between mature and elongation zones as well as between the varieties tested (Figure 4A,B). Highest and smallest peak and end fluxes of K^+^ were observed in Naso Nijo and CM 72, respectively, in the elongation root zone (Figure 4C,D). The same trend was found in the mature root zone for K^+^ peak fluxes, with a small difference in K^+^ end fluxes, where the highest flux was observed in another cultivar: Unicorn (Figure 4E,F). Ca^2+^ peak flux responses varied among cultivars (Figure 5A,B), with the highest and smallest Ca^2+^ fluxes observed in SYR 01 and Gairdner in elongation zone (Figure 5C), and Naso Nijo and ZUG 403 in mature zone (Figure 5D).

We then used a quantitative scoring system [53] to correlate the magnitude of measured flux responses with the salinity tolerance of each genotype. The overall salinity tolerance of barley was quantified as a damage index score ranging between 0 and 10, with 0 representing most tolerant and 10 representing most sensitive variety (Table 1). Peak and end flux values of K^+^ and Ca^2+^ were then plotted against respective tolerance scores. A significant (*p* < 0.05) positive correlation was found between H_2_O_2_-induced K^+^ efflux (Figure 4I,J), the Ca^2+^ uptake (Figure 5F), and the salinity damage index score in the mature root zone. At the same time, no correlation was found in the elongation zone for either K^+^ (Figure 4G,H) or Ca^2+^ flux (Figure 5E).

### 2.3. Genotypic Variation in H_2_O_2_-Induced Ca^2+^ and K^+^ Fluxes in Wheat

**Figure 6:**
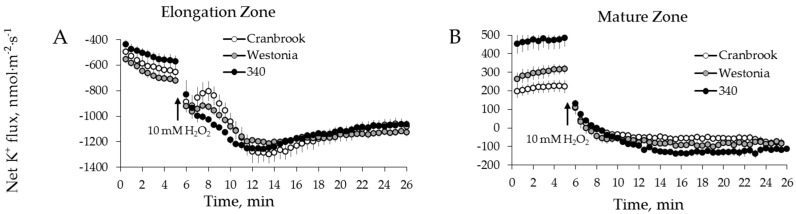

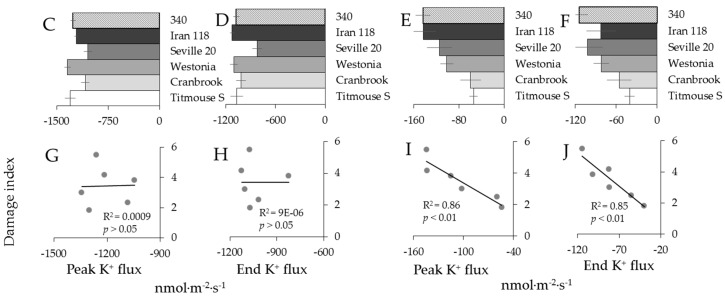
Kinetics of K^+^ fluxes from three representative wheat varieties in response to 10 mM H_2_O_2_ treatment from both root elongation zone (**A**) and mature zone (**B**). Error bars are means ± SE (*n* = 6–8). (**C**,**D**,**G**,**H**) Peak (**C**) and end (**D**) K^+^ fluxes of six wheat varieties in response to 10 mM H_2_O_2_ and their correlation with damage index (**G**,**H**, respectively) in root elongation zone. (**E**,**F**,**I**,**J**) Peak (**E**) and end (**F**) K^+^ fluxes of six wheat varieties in response to 10 mM H_2_O_2_ and their correlation with damage index (**I**,**J**, respectively) in root mature zone.

**Figure 7:**
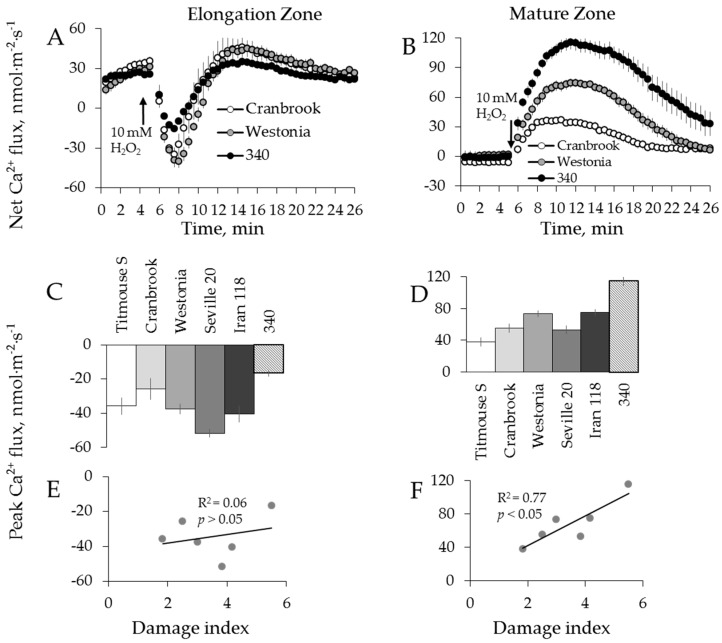
Kinetics of Ca^2+^ fluxes from three representative wheat varieties in response to 10 mM H_2_O_2_ treatment from both root elongation zone (**A**) and mature zone (**B**). Error bars are means ± SE (*n* = 6–8). (**C**,**E**) Peak Ca^2+^ fluxes (**C**) of six wheat varieties in response to 10 mM H_2_O_2_ and their correlation with damage index (**E**) in root elongation zone. (**D**,**F**) Peak Ca^2+^ fluxes (**D**) of six wheat varieties in response to 10 mM H_2_O_2_ and their correlation with damage index (**F**) in root mature zone.

Six wheat varieties contrasting in their salt tolerance were used to check whether the above trends observed in barley are also applicable to wheat species. Transient K^+^ and Ca^2+^ flux responses to 10 mM H_2_O_2_ in wheat were qualitatively identical to those measured from barley roots, in both zones (Figure 6A,B and Figure 7A,B). When peak and end flux values were plotted against the salinity damage index (Table 1, [54]), a strong positive correlation was found between H_2_O_2_-induced K^+^ (Figure 6E,F) and Ca^2+^ (Figure 7D) fluxes and the overall salinity tolerance (Table 1) in wheat root mature zone (*p* < 0.01 for Figure 6I,J; *p* < 0.05 for Figure 7F). Similar to barley, no correlation was found between salt damage index (Table 1) and the magnitude of ion flux responses (Figure 6C,D and Figure 7C) in the root elongation zone of wheat (Figure 6G,H and Figure 7E).

Taken together, the above results suggest that the H_2_O_2_-induced fluxes of Ca^2+^ and K^+^ in mature root zone correlate well with the damage index but no such correlation exists in the elongation zone.

### 2.4. Genotypic Variation of Hydroxyl Radical-Induced Ca^2+^ and K^+^ Fluxes in Barley

**Figure 8:**
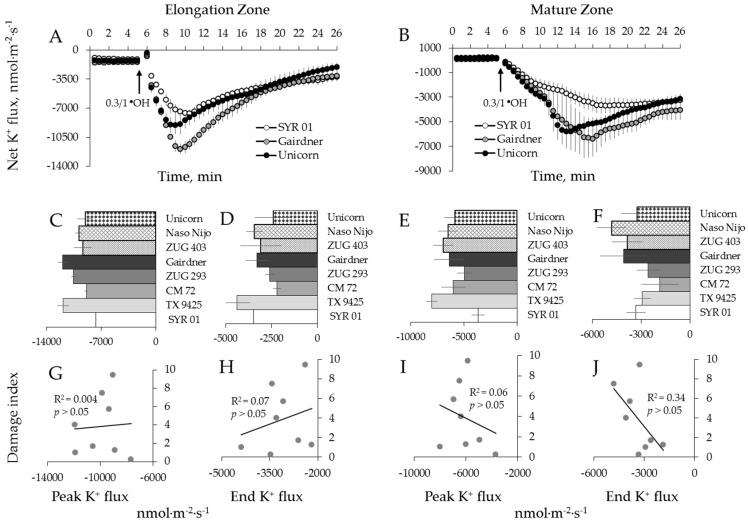
Kinetics of K^+^ fluxes from three representative barley varieties in response to 0.3/1 mM Cu^2+^/ascorbate mixture (^●^OH) treatment from both root elongation zone (**A**) and mature zone (**B**). Error bars are means ± SE (*n* = 6–8). (**C**,**D**,**G**,**H**) Peak (**C**) and end (**D**) K^+^ fluxes of eight barley varieties in response to ^●^OH and their correlation with damage index (**G**,**H**, respectively) in root elongation zone. (**E**,**F**,**I**,**J**) Peak (**E**) and end (**F**) K^+^ fluxes of eight barley varieties in response to ^●^OH and their correlation with damage index (**I**,**J**, respectively) in root mature zone.

Using eight barley varieties listed in Table 1, we then repeated the above experiments using a hydroxyl radical, the most aggressive ROS species of which can be produced during Fenton reaction between transition metal and ascorbate [55]. Hydroxyl radicals (^●^OH) were generated by applying 0.3/1.0 mM Cu^2+^/ascorbate mixture [48]. This treatment caused a dramatic K^+^ efflux (6–8 fold greater than the treatment with H_2_O_2_, data not shown), with fluxes reaching their peak efflux magnitude after 3 to 4 min of stress application in elongation zone and 7 to 13 min in the mature zone (Figure 8A,B). The mean peak values ranged from −3686 ± 600 to −8018 ± 536 nmol m^−2^·s^−1^ and from −7669 ± 27 to −11,930 ± 619 nmol·m^−2^·s^−1^, respectively, for the two zones (data not shown).

**Figure 9:**
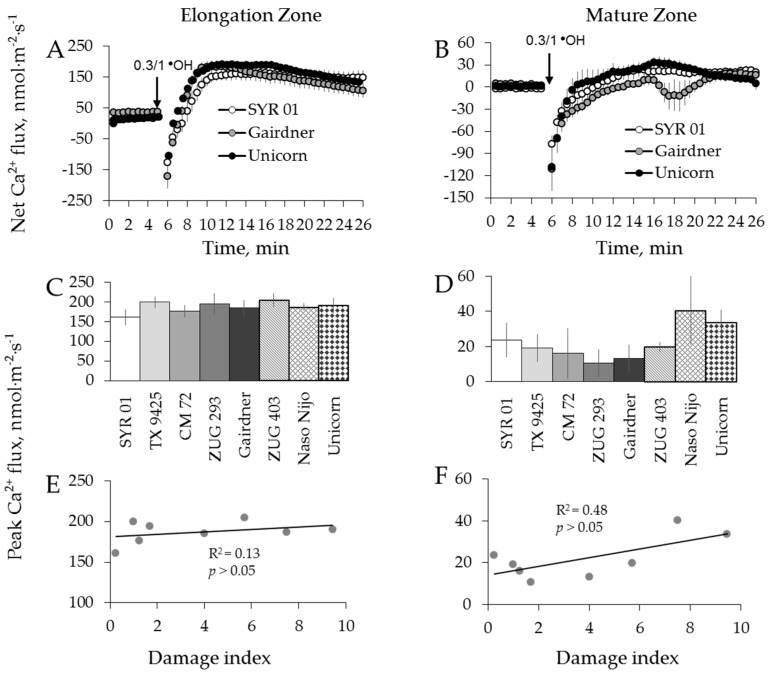
Kinetics of Ca^2+^ fluxes from three representative barley varieties in response to 0.3/1 mM Cu^2+^/ascorbate mixture (^●^OH) treatment from both root elongation zone (**A**) and mature zone (**B**). Error bars are means ± SE (*n* = 6–8). (**C**,**E**) Peak Ca^2+^ fluxes (**C**) of eight barley varieties in response to ^●^OH and their correlation with damage index (**E**) in root elongation zone. (**D**,**F**) Peak Ca^2+^ fluxes (**D**) of eight barley varieties in response to ^●^OH and their correlation with damage index (**F**) in root mature zone.

Contrary to H_2_O_2_ treatment, a dramatic and instantaneous net Ca^2+^ efflux was observed in both zones immediately after application of ^●^OH-generation mixture to the bath (Figure 9A,B). This Ca^2+^ efflux was short lived, and net Ca^2+^ influx was measured after about 2 min from elongation and after 8 min from mature root zones, respectively (Figure 9A,B). No significant correlation between overall salinity tolerance (damage index, see Table 1) and either Ca^2+^ or K^+^ fluxes in response to ^●^OH treatment was found in either zone (Figure 8G,H,I,J and Figure 9E,F).

## 3. Discussion

ROS are the “dual edge swords” that are essential for plant growth and signaling when they are maintained at the non-toxic level, but that can be detrimental to plant cells when ROS production exceeds a certain threshold [38]. This is particularly true for the role of ROS in plant responses to salinity. Salt-stress induced ROS production is considered to be an essential step in triggering a cascade of adaptive responses including early stomatal closure [56]; control of xylem Na^+^ loading [57,58] and sodium compartmentalization [59]. At the same time, excessive ROS accumulation may have negative impact on intracellular ionic homeostasis under saline conditions. Of specific importance is ROS-induced cytosolic K^+^ loss that stimulates protease and endonuclease activity, promoting program cell death [23,29,30,60]. Thereby, ROS homeostasis is required to maintain ROS concentrations at basal level, to facilitate redox biology, and act in signaling processes [20,21,38]. This homeostasis implies an involvement of highly orchestrated ROS generating and scavenging systems [20,61,62]. Here we show that such homeostasis is highly plant tissue-specific and differs between various ROS species.

### 3.1. The Magnitude of the Hydroxyl Radical-Induced K^+^ and Ca^2+^ Fluxes Does Not Correlate With Salinity Stress Tolerance in Barley

Hydroxyl radicals (^●^OH) are considered to be very short-lived (half-life of 1 ns) and highly aggressive agents that are a prime cause of oxidative damage to proteins and nucleic acids, as well as lipid peroxidation during oxidative stress [63]. At physiologically relevant concentrations, they have the greatest potency to induce activation of Ca^2+^ and K^+^ channels, leading to massive fluxes of these ions across cellular membranes [23,48] with detrimental effects on cell metabolism. This is clearly demonstrated by our data, showing that ^●^OH–induced K^+^ efflux was an order of magnitude stronger compared with that induced by H_2_O_2_, for the appropriate variety and a root zone (e.g., Figure 4 and Figure 8). Due to their short life, they can diffuse over very short distances (<1 nm) [64] and thus are less suitable for the role of the signaling molecules. Importantly, ^●^OH cannot be scavenged by traditional enzymatic antioxidants, and control of ^●^OH level in cells is achieved via elaborated network of non-enzymatic antioxidants (e.g., polyols, tocopherols, polyamines, ascorbate, glutathione, proline, glycine betaine, polyphenols, carotenoids; reviewed by Bose et al. [16]). It was shown that exogenous application of some of these non-enzymatic antioxidants prevented ^●^OH-induced K^+^ efflux from plant cells [65] and resulted in an improved salinity stress tolerance [66,67,68]. Thus, an ability of keeping ^●^OH levels under control appears to be absolutely essential for plant survival under salt stress conditions, and all barley genotypes, studied in our work, appeared to possess this ability (although, most likely, by different means).

A recent study from our laboratory [69] has shown that higher sensitivity of the root apex to salinity stress (as compared to mature root zone) was partially explained by the higher population of ^●^OH-inducible K^+^-permeable efflux channels in this tissue. At the same time, root apical cells responses to salinity stress by a massive increase in the level of allantoin, a substance with a known ability to mitigate oxidative damage symptoms [70] and alleviate ^●^OH-induced K^+^ efflux from root cells [69]. This suggests an existence of a feedback mechanism that compensates hypersensitivity of some specific tissue and protects them against detrimental action of ^●^OH. From our data reported here, it appears that the same mechanism exists amongst diverse barley germplasm. Thus, from the practical point of view, the lack of significant correlation between ^●^OH-induced ion fluxes and salinity stress tolerance (Figure 8 and Figure 9) makes this trait not suitable for salinity breeding programs.

### 3.2. H_2_O_2_-Induced K^+^ and Ca^2+^ Fluxes in Cereals Correlate with their Overall Salinity Stress Tolerance But Only in Mature Zone

Earlier observations showed that salt sensitive barley varieties (with higher damage index) have higher K^+^ efflux in response to H_2_O_2_ compared to salt tolerant varieties [44,71]. In this study, we extrapolated these initial observations made on a few selected varieties to a larger number of genotypes. We have also shown that (1) the same trend is also applicable to wheat species; (2) larger K^+^ efflux is mirrored by the higher Ca^2+^ uptake in H_2_O_2_-treated roots; and (3) the correlation between salinity tolerance and H_2_O_2_-induced ion flux responses exists only in mature but not elongation root zone.

Over the last decade, an ability of various plant tissues to retain potassium under stress conditions has evolved as a novel and essential mechanism of salinity stress tolerance in plants (reviewed by Shabala and Pottosin [30] and Shabala et al. [72]). Reported initially for barley roots [73,74,75], a positive correlation between the overall salinity stress tolerance and the ability of a root tissue to retain K^+^ was later expanded to many other species (reviewed by Shabala [49]) and also extrapolated to explain the inter-specific variability in salinity stress tolerance [76,77,78]. In roots, this NaCl-induced K^+^ efflux is mediated predominantly by outward-rectifying K^+^ channels GORK that are activated by both membrane depolarization [79] and ROS [23], as shown in direct patch-clamp experiments. Thus, the reduced H_2_O_2_ sensitivity of roots of tolerant wheat and barley genotypes may be potentially explained by either smaller population of ROS-sensitive GORK channels, or by higher endogenous level of enzymatic antioxidants in the mature root zone. It is not clear at this stage if H_2_O_2_ is less prone to induce K^+^ efflux (e.g., root cells are less sensitive to this ROS) in salt tolerant plants or the “effective” H_2_O_2_ concentration in root cells is lower in salt-tolerant plants due to a higher scavenging or detoxification capacity. However, given the fact that the activity of major antioxidant enzymes has been shown to be higher in salt sensitive barley cultivars in both control and H_2_O_2_ treated roots [44], the latter hypothesis is less likely to be valid.

The molecular identity of ROS-sensitive transporters should be revealed in the future pharmacological and (forward) genetic experiments. Previously we have shown that H_2_O_2_-induced Ca^2+^ and K^+^ fluxes were significantly attenuated in Arabidopsis *Atann1* mutants and enhanced in overexpressing lines [80], making annexin a likely candidate to this role. Further, H_2_O_2_-induced Ca^2+^ uptake in Arabidopsis roots was strongly suppressed by application of 30 µM Gd^3+^, a known blocker of non-selective cation channels [81], and roots pre-treatment with either cAMP or cGMP significantly reduced H_2_O_2_-induced K^+^-leakage and Ca^2+^-influx [82], implicating the involvement of cyclic nucleotide-gated channels (one type of NSCC) [83].

The lack of the above correlation between H_2_O_2_-induced K^+^ efflux and salinity tolerance in the elongation root zone is very interesting and requires some further discussion. In recent years, a “metabolic switch” concept has emerged [49,63], which implies that K^+^ efflux from metabolically active cells may be a part of the mechanism inhibiting energy-consuming anabolic reactions and saving energy for adaptation and reparation needs. This mechanism is implemented via transient decrease in cytosolic K^+^ concentration and accompanied reduction in the activity of a large number of K^+^-dependent enzymes, allowing a redistribution of ATP pool towards defense responses [49]. Thus, high K^+^ efflux from the elongation zone in salt-tolerant varieties may be an important part of this adaptive strategy. This suggestion is also consistent with the observation that plants often respond to salinity stress by the increase in the GORK transcript level [78,84].

It should be also commented that salt tolerant varieties used in this study usually have lower grain yield under control condition [75,85], showing a classical trade-off between tolerance and productivity [86], most likely as a result of allocation of a larger metabolic pool towards constitutive defense traits such as maintenance of more negative membrane potential in plant roots [72] or more reliance on the synthesis of organic osmolytes for osmotic adjustment.

### 3.3. Reactive Oxygen Species (ROS)-Induced K^+^ Efflux is Accompanied by an Increased Ca^2+^ Uptake

Elevation in the cytosolic free calcium is crucial for plant growth, development, and adaptation. Calcium influx into plant cells may be mediated by a broad range of Ca^2+^-permeable channels. Of specific interest are ROS-activated Ca^2+^-permeable channels that form so-called “ROS-Ca^2+^ hub” [87]. This mechanism implies that Ca^2+^-activated NADPH oxidases work in concert with ROS-activated Ca^2+^-permeable cation channels to generate and amplify stress-induced Ca^2+^ and ROS signals [48,81,83,88]. This self-amplification mechanism may be essential for early stress signaling events as proposed by Shabala et al. [88] and may operate in the root apex, where the salt stress sensing most likely takes place [89]. In the mature zone, however, continues influx of Ca^2+^ may cause excessive apoplastic ^●^O_2_ production where it is rapidly reduced to H_2_O_2_. By interacting with transition metals (Cu^+^ and Fe^2+^) in the cell wall, the hydroxyl radicals are formed [63], activating K^+^ efflux channels. This may explain the observed correlation between the magnitude of H_2_O_2_-induced Ca^2+^ influx and K^+^ efflux measured in this tissue (Figure 4I,J, Figure 5F, Figure 6I,J and Figure 7F). This notion is further supported by the previous reports that in Arabidopsis mature root cell protoplasts hydroxyl radicals were proved to activate and mediate inward Ca^2+^ and outward K^+^ currents [48,81], while exogenous H_2_O_2_ failed to activate inward Ca^2+^ currents [48]. The conductance resumed when H_2_O_2_ was applied to intact mature roots [81]. This indicated that channel activation by H_2_O_2_ may be indirect and mediated by its interaction with cell wall transition metals [55,90].

### 3.4. Implications for Breeders

Despite great efforts made in plant breeding for salt tolerance in the past decades, only limited success was achieved [91,92,93]. It becomes increasingly evident that the range of the targeted traits needs to be extended, shifting a focus from those related to Na^+^ exclusion from uptake [11,94,95,96] to those dealing with tissue tolerance. The latter traits have become the center of attention of many researchers in the last years [97,98]. However, to the best of our knowledge, none of the previous works provided an unequivocal causal link between salinity-stress tolerance and ROS activation of root ion transporters mediating ionic homeostasis in plant cells. This gap in our knowledge was filled by the current study.

Taken together, our results indicate high tissue specificity of root ion flux response to ROS and suggest that measuring the magnitude of H_2_O_2_-induced net K^+^ and Ca^2+^ fluxes from mature root zone may be used as a tool for cell-based phenotyping in breeding programs aimed to improve salinity stress tolerance in cereals. The next step in this process will be a full-scale validation of the proposed method and finding QTLs associated with ROS-induced ion fluxes in plant roots.

## 4. Materials and Methods

### 4.1. Plant Materials and Growth Conditions

Eight barley (seven *Hordeum vulgare* L. and one *H. vulgare ssp. Spontaneum*) and six wheat (bread wheat, *Triticum aestivum*) varieties contrasting in salinity tolerance were used in this study. All seeds were acquired from the Australian Winter Cereal Collection, and the list of cultivars is shown in Table 1. Seeds were surface sterilized with ten-fold diluted commercial bleach for 10 min and then rinsed thoroughly with tap water. Seeds were grown in basic salt medium (BSM; 0.1 mM CaCl_2_ and 0.5 mM KCl, pH 5.6) in aerated hydroponic system in 24 h darkness at 24 ± 1 °C for 4 days. Seedlings with root length between 60 and 80 mm were used in experiments.

### 4.2. Ion-Selective Microelectrodes Preparation

Net ion fluxes were measured with ion-selective microelectrodes non-invasively using MIFE technique (University of Tasmania, Hobart, Australia) [99]. Blank microelectrodes were pulled out from borosilicate glass capillaries (GC150-10, 1.5 mm OD × 0.86 mm ID × 100 mm L, Harvard Apparatus, UK) using a vertical puller, then dried at 225 °C overnight in an oven and then silanized with chlorotributylsilane (282707-25G, Sigma-Aldrich, Sydney, NSW, Australia). Silanized electrode tips were flattened to a diameter of 2–3 µm and backfilled with respective backfilling solutions (200 mM KCl for K^+^ and 500 mM CaCl_2_ for Ca^2+^). Electrode tips were then front-filled with respective commercial ionophore cocktails (Cat. 99311 for K^+^ and 99310 for Ca^2+^, Sigma-Aldrich,). Filled microelectrodes were mounted in the electrode holders of the MIFE set-up and calibrated in a set of respective calibration solutions (250, 500, 1000 µM KCl for calibrating K^+^ electrode and 100, 200, 400 µM CaCl_2_ for calibrating Ca^2+^ electrode) before and after measurements. Electrodes with a slope of more than 50 mV per decade for K^+^ and more than 25 mV per decade for Ca^2+^, and correlation coefficients of more than 0.9990 have been used.

### 4.3. Ion Flux Measurements

Net fluxes of Ca^2+^ and K^+^ were measured from mature (2–3 cm from root apex) and elongation (1–2 mm from root apex) root zones. To do this, plant roots were immobilized in a measuring chamber containing 30 ml BSM solution and left for 40 min for adaptation prior to the measurement. The calibrated electrodes were co-focused and positioned 40–50 µm away from the measuring site on the root before starting the experiment. After commencing, a computer-controlled stepper motor (hydraulic micromanipulator) moved microelectrodes 100 µm away from the positioned site and back in a 12 s square-wave manner to measure electrochemical gradient potential between two positions. The CHART software was used to acquire data [99,100] and ion fluxes were then calculated using the MIFEFLUX program [99].

### 4.4. Experimental Protocols for Microelectrode Ion Flux Estimation (MIFE) Measurements

Two types of ROS were tested—hydrogen peroxide (H_2_O_2_) and hydroxyl radicals (^●^OH). A final working concentration of H_2_O_2_ in BSM was achieved by adding H_2_O_2_ stock to the measuring chamber. As the half-life of H_2_O_2_ in the absence of transition metals is of an order of magnitude of several (up to 10) hours [101], and the entire duration of our experiments did not exceed 30 min, one can assume that bath H_2_O_2_ concentration remained stable during measurements. A mixture of copper/sodium ascorbate (Cu/A, 0.3/1.0 mM) was used to generate ^●^OH [48]. The measuring solution containing 0.5 mM KCl and 0.1 mM CaCl_2_ was buffered with 4mM MES/Tris to achieve pH 5.6. Net Ca^2+^ and K^+^ fluxes were measured from mature and elongation zones of a root for 4 to 5 min to ensure the stability of initial ion fluxes. Then a stressor (either H_2_O_2_ or ^●^OH) was added to the bath and Ca^2+^ and K^+^ fluxes were acquired for another 20 min. The first 30–60 s after adding the treatment solution (H_2_O_2_ or Cu/A mixture) were discarded during data analyses in agreement with the MIFE theory that requires non-stirred conditions [99].

### 4.5. Quantifying Plant Damage Index

The extent of plant salinity tolerance was quantified by allocating so-called “damage index score” to each plant. The use of such damage index is a widely accepted practice by plant breeders [53,54,102]. This index is based on evaluation of the extent of leaf chlorosis and plant survival rate and relies on the visual assessment of plant performance after about 30 days of exposure to high salinity. The score ranges between 0 (no stress symptoms) and 10 (completely dead plant), and it was shown before that the damage index score correlated strongly with the grain yield under stress conditions [102].

### 4.6. Statistical Analysis

Statistical significance of mean values was determined by the standard Student’s *t*-test at *p* < 0.05 level.

## Figures and Tables

**Table 1:** List of barley and wheat varieties used in this study. Scores represent quantified damage degree of cereals under salinity stress, reported as damage index score from 0 to 10.

Barley				Wheat			
Tolerant		Sensitive		Tolerant		Sensitive	
Varieties	Score	Varieties	Score	Varieties	Score	Varieties	Score
SYR 01	0.25	Gairdner	4.00	Titmouse S	1.83	Seville 20	3.83
TX 9425	1.00	ZUG 403	5.75	Cranbrook	2.50	Iran 118	4.17
CM 72	1.25	Naso Nijo	7.50	Westonia	3.00	340	5.50
ZUG 293	1.75	Unicorn	9.50				

0—highest overall salinity tolerance; 10—lowest level of salt tolerance. Data collected from our previous study from Wu et al. [53,54].

## Abbreviations

ROS	Reactive Oxygen Species
SOS	Salt Overly Sensitive
HKT	High-affinity K^+^ Transporter
NSCCs	Non-Selective Cation Channels
GORK	Guard cell Outward Rectifying K^+^ channel
PCD	Programmed Cell Death
AO	Antioxidant
MIFE	Microelectrode Ion Flux Estimation
QTL	Quantitative Trait Locus
BSM	Basic Salt Medium
Cu/A	Copper/Ascorbate
